# The Promises and Realities of Integration in Synthetic Biology: A View From Social Science

**DOI:** 10.3389/fbioe.2020.622221

**Published:** 2021-01-07

**Authors:** Lucy Carter, Aditi Mankad

**Affiliations:** ^1^CSIRO Synthetic Biology Future Science Platform, Brisbane, QLD, Australia; ^2^CSIRO Land & Water, Brisbane, QLD, Australia

**Keywords:** science integration, social science, interdiscipinarity, synthetic biology, responsible innovation

## Abstract

Discussions about the responsible advancement of synthetic biology science are at fever pitch. Commentators from across the globe are calling for greater integrated science investments and more inclusive governance processes in the development and implementation of these potentially disruptive technologies. We take stock of the promises and realities of science integration by sharing our experiences of embarking on this very challenge in Australia. We conclude by offering suggestions for bringing about the enabling conditions for improved integration across the natural and social sciences. Four key actions are articulated to help pivot synthetic biology toward a more integrated scientific endeavor: (a) formalizing inclusivity from inception to project conclusion; (b) valuing differing philosophical positions as a strength rather than a barrier; (c) accepting that integration takes persistence and communication but is immensely rewarding; and (d) promoting meaningful interactions, such as pursuing joint opportunities, co-designing and co-publishing research. We argue that these actions are key enablers for realizing science integration in synthetic biology.

Synthetic biology offers scientists the opportunity to modify living systems by applying novel genetic techniques which have the potential to address pressing environmental and industrial challenges (Lai et al., 2019; Delborne et al., 2020). To realize this impact, deep and genuine science integration between disparate disciplines (and their institutions) will be required. The integration of biological and social sciences, for example, allows for comprehensive examination of problems and their solutions using distinct but complimentary scientific paradigms. Specifically, integration allows biological solutions to be re-cast in a social context – essentially enabling more sustained real-world impact beyond academic circles (Viseu, 2015; National Academies of Sciences, Engineering, and Medicine (NASEM), 2016; Emerson et al., 2017; Kofler et al., 2018; French, 2019). Our appraisal of practicing integration seeks to deepen and extend emerging dialogue between the sciences and their institutions (e.g., Nature News, 2015), to enable more impactful outcomes in the synthetic biology domain.

## The Need for Integration

Despite the need for varied scientific perspectives along the path from translating molecular science to technology implementation, functional and meaningful integration in the fields of synthetic biology and social sciences remains a challenge. While bioscience research tends to forge ahead at a rapid pace, often progressing in isolation from other disciplines (Esvelt and Gemmell, 2017), the social sciences and humanities have been slower to inform, shape and support synthetic biology innovations compared to nanotechnology applications, for example (Shapira et al., 2015). When social science research is included, it can be *ad hoc* despite being heralded as “critical” to the success of an innovation in modern disruptive technology narratives (Calvert and Martin, 2009; Trump et al., 2018). This often results in social science contributions being limited in scope and undertaken only once initial bioscience development is already in full-swing (Taylor and Woods, 2020). Adding to this broader challenge, shrinking research budgets and timelines can render allocation of financial resources for integration tokenistic, hindering genuine integration.

As societies face more complex environmental, health, and industrial challenges, and governments and the private sector turn to the promises of synthetic biology innovations, a global shift must occur in the way that science is organized and valued (Taebi et al., 2014; Taylor and Woods, 2020). Where once the biosciences were the key to scientific progress, equally recognizing the role of allied sciences in bringing those technologies to bear will be critical to realizing real-world impact. Recent efforts to overcome historical barriers to interdisciplinary science are emerging, observable through the development of frameworks such as Responsible (Research and) Innovation (RRI/RI) where normative principles and approaches guide science decision-making and planning (Owen et al., 2012; von Schomberg, 2013). Aspirational in vision, “responsible innovation means taking care of the future through collective stewardship of science and innovation in the present” (Stilgoe et al., 2013: 1570). Science integration (or *genuine* interdisciplinary collaboration) is a necessary, yet small step along this process. However, a number of structural, institutional and practical hurdles continue to make integration challenging. Further complicating the goal of seamless integration are the lingering legacy drivers for pursuing science integration in the area of synthetic biology and the starting point from where some of these motivations may have originated.

## Historical Hurdles

A primary driver for the integration of social and biological sciences in the field of synthetic biology has been a collective desire to “avoid the GM mistakes of the past,” where science and industry largely neglected to consider the social dimensions of proposed genetic modification (GM) of agricultural commodities (Calvert, 2013; Fisher et al., 2013). These omissions led to outrage in certain sections of society, diminishing trust in scientists and their organizations more broadly. The promulgation of negative messaging toward biotechnology culminated in a global cultural phenomenon where the mere mention of “GM” can render a technology or product inert and its developers branded social and institutional pariahs.

To overcome some of these past mistakes, science institutions have more earnestly promoted a holistic approach to solving some of the world's greatest challenges by requiring funding applicants to identify the ethical and social considerations of the work they intend to conduct. However, there is rarely a sufficiently strong accountability mechanism that requires research teams to follow-through on addressing the social impacts identified.

For some research groups striving for broader impact, stimulating science integration can equate to obtaining “social licence” for a particular application and its adoption. Often touted as the holy grail for achieving science impact, the social license aspiration can invoke a transactional paradigm of engagement with the anticipated end result being a rubber stamp of approval. Without sufficient oversight from independent engagement practitioners, a social license agenda can shift an intended engagement goal to a campaign of (re)educating the public on the benefits and impacts of a proposed novel application. In the synthetic biology context, this framing not only limits the exploration and identification of other complex sociocultural factors, but it is also inappropriate (Delborne et al., 2020). If we accept the definition of social license as representing an “unwritten social contract” for approval of novel genetic biotechnologies more generally, as described by Lacey and Lamont (2014) and others, then it would appear that we are too late (Lacey and Lamont, 2014; Moffat and Zhang, 2014). Laboratories around the world are already editing the genomes of organisms, the end products of which are already commercially available without a social license (e.g., sugar beet in the US, cotton in Australia). Due to the rapid pace of genome editing and synthetic biology development, what may have gained social licence 5 years ago in a given context may not have ongoing support or be generalisable across contexts. Neither a desire to avoid the GM mistakes of the past, nor the pursuit of social license as it is currently conceptualized, is likely to culminate in genuine integration.

Social science has successfully been used to “smooth the way” for more inclusive deliberations about risks, for identifying pathways for regulation, and for pursuing public acceptance for novel applications (Calvert and Frow, 2013; Jones, 2014). In our experience, these discussions are useful for building rapport amongst scientists from divergent disciplines and can build shared understandings of methodological differences and operational challenges. However, a deeper integration of science has the potential to create space for discussions about the broader, bigger, science questions such as *How best do we engage a largely uncertain public about conversations in science? How do we determine public good science? How can multiple science disciplines be on equal footing at each stage of the science planning process?* Beyond the more intellectually stimulating research questions, there are also fundamental social and ethical imperatives to pursue scientific advances in a balanced and procedurally just manner (Jones, 2014).

## Barriers to Meaningful Integration: *in-situ* Reflections

Our professional experience with pursuing integration science has provided us with insights regarding some of the challenges that this approach faces. As social scientists working in applied science, we rarely enjoy the certainty of a well-trodden research path. Not unlike the natural sciences, we are required to defend our chosen methodologies, our theoretical positions, and the ethical dilemmas that cross-disciplinary integration raises. Where the natural sciences are widely recognized as comprising a multitude of disciplines, the social and behavioral sciences are regularly treated as one discipline with finite value, despite including at least seven distinct disciplines and many more sub-disciplines. In addition, an implicit assumption in cross-disciplinary collaborations remains that social scientists should drive, and are indeed responsible for driving an integration agenda (Balmer et al., 2016).

Other examples of “disciplinary encounters” we have experienced in our quest to integrate include: persuading colleagues that qualitative approaches can be both rigorous and valuable; that *all* scientific data is socially constructed and; that the deliberate advocacy of certain synthetic biology applications by social scientists places *all sciences* at professional risk. While increasingly the deployment of the social sciences is considered essential, it is often characterized as “in service to” the biosciences rather than a legitimate research endeavor in and of itself (Balmer et al., 2016). Scientists must *collectively* work hard to ensure the value proposition for social science is equally regarded in the broader synthetic biology agenda particularly if we aspire to innovate responsibly, and especially if science is funded by governments to deliver public goods.

## Lessons for Social Scientists, and Others

The promise of integration also requires social scientists to self-reflect on our role in poor integration efforts. As social scientists, we might make confident statements about how effective public engagement ought to be conducted, for example, perhaps without a clear sense of how that information translates to actionable outcomes. This can often leave bioscientists frustrated by a lack of guidance and uncertainty about how best to integrate social data into biological methods in a meaningful way. It is also true that social scientists can retreat to more rigid theoretical positions that leave no room for reflexive integration. We must acknowledge (uneasily) that it is entirely possible that some synthetic biology advancements do not need an integrated scientific approach and can be brought to bear without societal engagement because of their immense market pull and/or proximal relationships with the end user (Balmer et al., 2016).

An uncomfortable reality is that traditional and accepted knowledge within the social and behavioral sciences applies largely Western framings of science, where scholarship has typically neglected traditional knowledge systems developed by others, particularly indigenous and local communities (Mazzocchi, 2006). A more integrated scientific approach should include an acknowledgment of the multiple knowledge systems needed to integrate for optimal science impact. Genuine integration requires mutual respect for other disciplinary and interdisciplinary approaches that hold a value proposition equal to one's own home discipline; and trust that other disciplines have the technical know-how to understand the various moving parts from their own distinctive lens.

In practice, there are key challenges facing both social and natural scientists who choose to engage with integrative science. These include a loss of disciplinary purity and loss of expertise which can lead to difficulties in academic progression and; perceptions of dual interests and bias, where scientists within the same organization develop the foundational technology and a separate arm of the organization evaluates the social and ethical implications of that technology. A more difficult pathway to successfully publishing in high impact scientific journals is also a cost to scientists more broadly as a result of integration, especially for those who choose to work at the boundaries of traditional disciplinary silos or at the interface between research and practice.

Another key challenge is that of language and culture, where a misunderstanding of terminology across disciplines can lead to frustration or unintentional offense. Understanding that integration is a two-way process of cultural change for all scientists, and that cultural changes take time and must be championed with sufficient motivation and effort, is a key lesson.

## Integration as a Means to Maximize Science Impact

While lessons from the effective and meaningful integration of socially relevant data into bioscience design processes are yet to fully emerge, there are clear signals from previous poor integration and engagement efforts which can inform the success of synthetic biology innovations. To conclude, we offer our suggestions and actions for enabling integration across natural and social sciences to improve the impact of synthetic biology endeavors.

### All Scientists Need a Seat at the Table

While current methods of integrating social science *ad hoc* into biological and natural science projects can be effective and can yield some science impact, the involvement of social and ethical considerations during project inception through to its conclusion is more valuable. The positioning of institutional (and organizational) structures which embed and enable integration are those likely to yield more sustainable results. For example, incentivising and rewarding co-publication efforts, is one method of enabling integration early. As is requiring collaboration across disciplinary divides, as a mechanism of funding or commencement approval.

A common fear we have encountered is that involving public stakeholders “too early” may derail projects or place an artificial ceiling on key exploratory science before projects even get off the ground. We question whether this fear is well-founded and whether investment in synthetic biology research within impact-driven institutions should indeed be pursued if the public were to reveal ambivalence or disapproval, given that much of the exploratory research in synthetic biology is publicly funded. Moreover, highly successful proof of concept technologies within a research institute or academic setting can be positioned as having enormous triple-bottom line impact value, yet broader socioeconomic conditions are not adequately considered or planned for.

### Mutual Value and Autonomy

A fundamental hurdle to true science integration in synthetic biology is the valuing of unique contributions of other disciplines in helping to write the complex story that is innovation. We urge research leaders and administrators to resist micromanaging alternative science output when methodological approaches do not fit the “experimental science” mold or when non-traditional knowledge processes might be more suitable. All scientists have had comparable training for their craft and differing philosophical positions are a strength rather than a barrier. An environment where scientists from different disciplines are trusted and governed accordingly fosters intellectual freedom which has the potential to yield impactful outputs on a global scale.

More broadly, the value of both biosciences and social sciences is no more obvious than when a technology is conceptualized as a “game changer” in the laboratory, only to discover that there is no social “pull” for a technological solution in its application. Recognizing the mutual value of co-developing an innovative solution, or revolutionizing an industry for a local community, will help to foster trust and redress any perceived imbalances across disciplines.

### Go All In

Integration science is awkward at first, but it does get easier – regular communication, persistence and the passing of time have helped to build bridges. Be prepared to feel uncomfortable and be prepared not to like what you find. As with all new ventures, there are risks to be mindful of and risks to be taken – not all of which are negative. In our experience, reaching out to different disciplines can be immensely rewarding and there are potential benefits to be realized, such as greater funding opportunities and clearer pathways to impact.

### Seal the Deal With Meaningful Interaction

Opportunities that help integration are not always obvious. Pursuing collaborative science or strategic planning activities are steps that lay the foundations for integration. We have actively pursued opportunities to genuinely collaborate across disciplinary and application domains. Our experience of co-designing science communication materials for use in research instruments has been a positive one. We are currently co-authoring research publications with cross-disciplinary colleagues and have aspirations to jointly develop impact pathways for future science planning.

The funding of science to be more co-dependent such that investments in synthetic biology, or other novel science areas, formally require inclusion of integrated work packages which facilitate cross-disciplinary science would aid integration efforts. This would help bridge the gap between merely identifying possible social and ethical issues and extending that requirement to tackling the impacts identified. Such a fundamental shift toward integration in the funding of science could better realize science impacts.

## Final Thoughts

There are key levers for realizing science integration. Presently there is limited exploration in the literature of how synthetic biology solutions might manifest in a social world. There are paths we know will not serve the synthetic biology research community well such as adopting transactional approaches to public engagement. Collectively, we must resist extrapolating previous research findings conducted during the GM era. We should also resist asking questions of stakeholders using generalized concepts like “synthetic biology” and assume that responses are valid or generalisable for all types of synthetic biology research. The factors driving public perceptions of industrial solutions are likely to be very different from those influencing attitudes toward environmental or personalized health solutions (Mohr et al., 2007).

Science integration demands an authentic commitment from both researchers and funders to effectively resource and conceptually engage. Simply setting aside a small proportion of the budget to fund social license activities is not enough. Genuine collaboration, a core feature of integration, requires a shift in how science is planned and rewarded. It requires organizations to consider how programs and projects are designed and resourced, and how the sciences *talk* to each other. An actionable commitment to integration is much more likely to advance responsible science innovation.

## Data Availability Statement

The original contributions presented in the study are included in the article/supplementary material, further inquiries can be directed to the corresponding author/s.

## Author Contributions

Both authors listed have made a substantial, direct and intellectual contribution to the work, and approved it for publication.

## Conflict of Interest

The authors declare that the research was conducted in the absence of any commercial or financial relationships that could be construed as a potential conflict of interest.
